# Universal fractality of morphological transitions in stochastic growth processes

**DOI:** 10.1038/s41598-017-03491-5

**Published:** 2017-06-14

**Authors:** J. R. Nicolás-Carlock, J. L. Carrillo-Estrada, V. Dossetti

**Affiliations:** 1 Instituto de Física, Benemérita Universidad Autónoma de Puebla, Puebla, 72570 Mexico; 2 CIDS-Instituto de Ciencias, Benemérita Universidad Autónoma de Puebla, Puebla, 72570 Mexico

## Abstract

Stochastic growth processes give rise to diverse and intricate structures everywhere in nature, often referred to as fractals. In general, these complex structures reflect the non-trivial competition among the interactions that generate them. In particular, the paradigmatic Laplacian-growth model exhibits a characteristic fractal to non-fractal morphological transition as the non-linear effects of its growth dynamics increase. So far, a complete scaling theory for this type of transitions, as well as a general analytical description for their fractal dimensions have been lacking. In this work, we show that despite the enormous variety of shapes, these morphological transitions have clear universal scaling characteristics. Using a statistical approach to fundamental particle-cluster aggregation, we introduce two non-trivial fractal to non-fractal transitions that capture all the main features of fractal growth. By analyzing the respective clusters, in addition to constructing a dynamical model for their fractal dimension, we show that they are well described by a general dimensionality function regardless of their space symmetry-breaking mechanism, including the Laplacian case itself. Moreover, under the appropriate variable transformation this description is universal, i.e., independent of the transition dynamics, the initial cluster configuration, and the embedding Euclidean space.

## Introduction

Found everywhere in nature, the intricate structures generated by fractal growth usually emerge from non-trivial self-organizing and self-assembling pattern formation^1–4^. One striking feature of these systems is the morphological transition they undergo as a result of the interplay between entropic and energetic processes in their growth dynamics, that ultimately manifest themselves in the geometry of their structure^5^. It is here where, despite their complexity, great insight can be obtained into the fundamental elements of their dynamics from the powerful concepts of fractal geometry^6, 7^. Such is the case of the Laplacian growth or Dielectric Breakdown Model (DBM)^8, 9^ that has importantly contributed to our understanding of far-from-equilibrium growth phenomena, to such an extent that seemingly unrelated patterns found in nature, such as river networks or bacterial colonies, are now understood in terms of a single framework of complex growth^10, 11^. Nonetheless, a complete scaling theory of growth far-from-equilibrium has been missing and, consequently, a comprehensive description of the fractality of systems that exhibit fractal to non-fractal morphological transitions as well^7, 12^.

**Figure 1:**
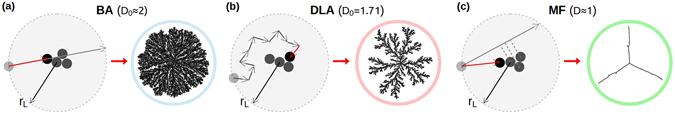
Fundamental aggregation models. After being launched into the system from *r*_*L*_ with uniform probability, particles (**a**) follow straight-line trajectories before aggregation in BA, (**b**) perform a random walk in DLA, and (**c**) get radially attached to the closest particle in the cluster as a result of an infinite-range radial interaction in MF aggregation. The last one is particle-path independent and its morphological characteristics emerge solely from this long-range interaction as opposed to the stochastic BA and DLA models. Characteristic clusters for each model with their respective fractal dimension are shown.

Laplacian theory assumes that, in the absence of long-range interactions, the growth probability at a given point in space, *μ*, is generated by the spatial variation of a scalar field, *ϕ*, i.e., $\mu \propto |\nabla \varphi |$. One example of such processes is the paradigmatic diffusion-limited aggregation (DLA) model, where particles performing a random walk aggregate one-by-one to form a cluster, starting from a seed particle (see Fig. 1). In particular, the structure that emerges from this process can be described by a single fractal dimension, *D*, only dependent on the Euclidean dimension of its embedding space, *d*. For the off-lattice two-dimensional (*d* = 2) case, the corresponding fractal dimension has been widely reported to have a value *D* = 1.71^12^. Furthermore, the theory has been extended to consider a more general and interesting growth process^13–17^ in which the mean-square displacement of the particles’ trajectories, as the control parameter, generates a continuous morphological transition that can be neatly described through the fractal dimension of the walkers’ trajectories, *d*_*w*_. This transition goes from a compact cluster with *D* = *d* for *d*_*w*_ = 1, as expected for ballistic-aggregation (BA), to the DLA fractal for *d*_*w*_ = 2^18^ (see Fig. 1).

However, one of the most challenging aspects of the theory arises when the growth is not purely limited by diffusion, e.g., when it takes place under the presence of long-range attractive interactions, where strong screening and anisotropic effects must be taken into account. In this case, the growth probability has been generalized to the form $\mu \propto {|\nabla (\varphi )|}^{\eta }$, where *η* is a positive real number associated with all the effects coming from screening and anisotropy^8, 9^. For a given embedding Euclidean space of dimension *d*, this process generates a characteristic morphological transition as function of *η*, that goes from an initial compact structure with *D* = *d* for *η* = 0, associated with Eden clusters, passing through DLA fractals for *η* = 1, to a linear cluster with *D* = 1 as *η* → ∞. In addition, it has been suggested that the transition to the last one occurs at a critical value $\eta \approx 4$, where this criticality is understood in terms of the fractality of the system, i.e., the value of *η* at which $D\approx 1$ ^19–21^. Nonetheless, the use of the fractal dimension *D* as an order parameter, able to describe the criticality of these transitions, still needs some clarification.

One of the best analytical results to describe the fractality of transitions such as BA-DLA and the one associated with the DBM, is the generalized Honda-Toyoki-Matsushita mean-field equation^22–24^:

1 $${D}_{{\rm{MF}}}(d,{d}_{w},\eta )=\frac{{d}^{2}+\eta ({d}_{w}-1)}{d+\eta ({d}_{w}-1)}.$$

For *η* = 1, this equation gives a good description of the BA-DLA transition, whereas for *d*_*w*_ = 2, it is intended to describe the DBM in any embedding dimension *d*. In particular, for the case *d* = 2, this expression provides a good qualitative description of the fractality of the DBM transition, however, due to its mean-field limitations, it fails to precisely reproduce the reported numerical results for *D*(*η*)^25, 26^ (see Table 1). For example, it underestimates the known fractal dimension of DLA clusters for *d* = 2, nor does it clearly predict any criticality as suggested. As far as we know, there is not any analytical result able to fully describe the scaling or fractality of these and similar processes^7, 12^.

**Table 1:** Fractal dimensions of the DBM.

Data source	*η* = 0.5	*η* = 1	*η* = 2	*η* = 3	*η* = 4	*η* = 5
Niemeyer, *et al*.^8^ *	1.89 ± 0.01	1.75 ± 0.02	1.6			
Hayakawa, *et al*.^24^ *	1.79 ± 0.01		1.47 ± 0.03			
Pietronero, *et al*.^9^	1.92	1.70	1.43			
Somfai, *et al*.^25^		1.71	1.42	1.23		
Amitrano^26^	1.86	1.69	1.43	1.26	1.16	1.07
Hastings^21^			1.433	1.263	1.128	1.068
			1.426	1.264	1.090	1.030
			1.435	1.262	1.078	1.025
			1.452	1.243	1.071	1.009
〈*D*(*η*)〉	1.89 ± 0.05	1.70 ± 0.01	1.44 ± 0.02	1.25 ± 0.01	1.11 ± 0.04	1.04 ± 0.03
*D*_*MF*_(*η*)	1.80	1.67	1.50	1.40	1.33	1.29
*D*(*η*) (theoretical)	1.88	1.71	1.37	1.16	1.06	1.02

The first section of the table summarizes the fractal dimensions reported for the two-dimensional version of the model. The second section of the table is filled with the average fractal dimension 〈*D*(*η*)〉, obtained as the arithmetic mean over each column with its corresponding standard deviation, the mean-field theory and the analytical result obtained here (last row). Data sources marked with an asterisk are not considered in the average due to limitations in their simulations. The error in the reported measurements is shown if available.

In this work, in order to clarify these aspects of the Laplacian theory, as well as to establish a possible general framework to analyze more complex morphological transitions in stochastic growth processes, we present a dynamical model that addresses the fractality of these transitions. Moreover, it is able to precisely describe the observed scaling from an initial non-fractal/fractal configuration, towards a linear one, in terms of a single function associated to all the dynamical growth mechanisms present in the system.

The validity of our model is tested twofold. First, under a Monte Carlo approach to fundamental particle aggregation dynamics, we combined three basic *off*-*lattice* particle-cluster aggregation models, the DLA, BA, and an infinite-range mean-field (MF) attractive model, in order to generate BA-MF and DLA-MF (fractal to non-fractal) morphological transitions as function of a mixing parameter *p*. For these transitions, that are able to reproduce all the main morphologies of fractal growth, the scaling of the generated clusters is measured locally and globally for different values of *p* using two standard methods: the two-point density correlation function and the radius of gyration. In second place, we apply the model to the scaling of the DBM (by making use of data already available in the literature, given in Table 1). In particular, we show how our model contains equation (1) as a special case within its first-order approximation. In both cases, we show that the solution is able to describe the measured scaling of these clusters, i.e., *D*(*p*) and *D*(*η*), for the BA/DLA-MF and DBM transitions, respectively. Finally, we show how all of the data collapse to single universal curves, independently of any initial configuration, growth process, and embedding Euclidean space.

## Results

### Generalized dimensionality function

Despite the complexity of the transitions mentioned above, a simple model can be established to describe their fractality or scaling. This is done by considering that the fundamental dynamical elements that drive the fractal growth are mainly two: *stochastic* and *energetic*. As previously discussed, when the growth dynamics is purely driven by stochastic processes, as in the case of DLA (*η* = 1) or BA (similar to *η* = 0), the resulting structure is either a fractal (DLA) or a compact fat-fractal (BA) with *D* ≤ *d*. However, when an energetic element is introduced in the growth dynamics, the fractal dimension of the clusters decreases; for example, *D* → 1 as *η* → ∞ in the DBM.

As such, in the most general case, we consider that these transitions start with clusters produced by purely stochastic dynamics, with *D* = *D*_0_, where *D*_0_ stands for the fractal dimension of the clusters in this regime. Further on, as energetic mechanisms that drive spatial symmetry-breaking increase, such as strong non-linear interactions, for example, these clusters collapse to linear structures. Let us also consider that all the information regarding the effects of stochastic and energetic growth-dynamics is encoded in an effective control parameter ${\rm{\Phi }}$, allowing us to define a generalized dimensionality function $D({\rm{\Phi }})$. In this way, as a function of ${\rm{\Phi }}$, we require that $D({\rm{\Phi }})={D}_{0}$ for ${\rm{\Phi }}=0$ and that $D({\rm{\Phi }})\to 1$ as ${\rm{\Phi }}\to \infty$ along the transition. Correspondingly, in terms of the co-dimension, $\hat{D}=D-1$, we would have $\hat{D}({\rm{\Phi }})={D}_{0}-1$ for ${\rm{\Phi }}=0$ and $\hat{D}({\rm{\Phi }})\to 0$ as ${\rm{\Phi }}\to \infty$. Additionally, for this kind of morphological transitions, it has been observed that the dependence of *D* on the control parameter is smooth and monotonically decreasing^21, 24, 26–29^. From this, since ${\rm{d}}D/d{\rm{\Phi }}={\rm{d}}\hat{D}/d{\rm{\Phi }}$ is satisfied, the most general solution for the scaling of the clusters is obtained from ${\rm{d}}\hat{D}/d{\rm{\Phi }}=-f(\hat{D})$, with $f(\hat{D}) > 0$. By expanding $f(\hat{D})$ as a Taylor series we have: ${\rm{d}}\hat{D}/d{\rm{\Phi }}\approx -[{f}_{0}+{f}_{1}\hat{D}+O({\hat{D}}^{2})]\approx -{f}_{0}-{f}_{1}\hat{D}$, where we have truncated the series up to the linear term as, again, we expect $\hat{D}$ to vary smoothly along the transition. Thus, by integrating on both sides of the equation for given and finite $\hat{D}$ and ${\rm{\Phi }}$, i.e., ${\int }_{{\hat{D}}_{0}}^{\hat{D}}d\hat{D}^{\prime} /({f}_{0}+{f}_{1}\hat{D}^{\prime} )={\int }_{0}^{{\rm{\Phi }}}d{\rm{\Phi }}^{\prime}$ and by taking into account the condition that $\hat{D}({\rm{\Phi }})\to 0$ as ${\rm{\Phi }}\to \infty$, we obtain:

2 $$D({\rm{\Phi }})\mathrm{=1}+({D}_{0}-1){e}^{-{\rm{\Phi }}},$$

where the constant *f*_1_ has been absorbed in the control parameter ${\rm{\Phi }}$.

Under the conditions stated above, equation (2) is the most general form for the fractality of clusters found in morphological transitions, driven by stochastic and energetic mechanisms. For a particular case, the effective parameter ${\rm{\Phi }}$ must still be found and is expected to depend on the parameters of a given system. As explained below, finding the correct ${\rm{\Phi }}$ is not trivial and special dynamical conditions over *D* will be required. Before considering a more general scenario, let us now show why this equation is suitable to characterize these systems by considering the DBM mean-field equation first.

The mean-field result given in equation (1) belongs to a special case of the family of equations given in (2). Starting with the first-order approximation in ${\rm{\Phi }}$ of equation (2), it follows that,

3 $${D}^{\mathrm{(1)}}({\rm{\Phi }})=1+\frac{{D}_{0}-1}{1+{\rm{\Phi }}}=\frac{{D}_{0}+{\rm{\Phi }}}{1+{\rm{\Phi }}}\mathrm{.}$$

Here, by setting *D*_0_ = *d* and from direct comparison with equation (1), one is able to observe that these expressions are equivalent, with ${\rm{\Phi }}$ being nothing but ${{\rm{\Phi }}}_{MF}=\eta ({d}_{w}-1)/d$. This approximate result makes the relation between the effective parameter ${\rm{\Phi }}$ and the actual parameter of the transition (in this case *η*) more evident and, for a given *d* (with *d*_*w*_ = 2), it exhibits a linear relation between the parameters, ${\rm{\Phi }}\propto \eta$, which, as stated before, does not provide the correct solution for *D* due to its mean-field nature^25, 26^. Thus, a more general function for ${\rm{\Phi }}(d,\eta )$ is still required.

There is one more condition that should be imposed over *D* in order to have a better and more general prescription for ${\rm{\Phi }}$. When the fractal dimension of a cluster goes from *D* = *D*_0_ ≤ *d* to *D* = 1 throughout the transition, due to the competition of the stochastic and energetic elements of the growth dynamics, two regimes can be clearly defined in the extremes. For this to happen, there will necessarily exist a regime change in between, where neither the stochastic nor the energetic mechanisms dominate. Regarding the behavior of *D*, let us consider that this change in regime is associated with the point where the second derivative of *D* with respect to its control parameter becomes zero. This is, if ${\rm{\Phi }}={\rm{\Phi }}({D}_{0},\zeta )$, where $\zeta$ is the parameter that controls the transition of a given system, then, there is an inflection point ${\zeta }_{{\rm{i}}}$ that satisfies, ${{{\rm{d}}}^{2}\hat{D}/{\rm{d}}{\zeta }^{2}|}_{\zeta ={\zeta }_{{\rm{i}}}}=0$. This *inflection* condition, from equations (2) and (3), translates to ${[{(d{\rm{\Phi }}/{\rm{d}}\zeta )}^{2}-{{\rm{d}}}^{2}{\rm{\Phi }}/{\rm{d}}{\zeta }^{2}]|}_{\zeta ={\zeta }_{{\rm{i}}}}=0$ and ${[2{(d{\rm{\Phi }}/{\rm{d}}\zeta )}^{2}-(1+{\rm{\Phi }}){{\rm{d}}}^{2}{\rm{\Phi }}/{\rm{d}}{\zeta }^{2}]|}_{\zeta ={\zeta }_{{\rm{i}}}}=0$.

For example, as it can be observed in the DBM mean-field case by identifying $\zeta$ as *η*, the relation between parameters is linear, i.e., ${{\rm{\Phi }}}_{MF}={\rm{\Lambda }}\zeta /{D}_{0}$ (with ${\rm{\Lambda }}={d}_{w}-1$ and fixed *D*_0_), making it impossible to define ${\zeta }_{{\rm{i}}}$, as the inflection condition cannot be satisfied. Therefore, we propose ${\rm{\Phi }}({D}_{0},\zeta )={\rm{\Lambda }}{\zeta }^{\chi }/{D}_{0}$ as a general *ansatz* for ${\rm{\Phi }}$, where ${\rm{\Lambda }}$ and *χ* are two positive real numbers that are associated with the strength of the screening/anisotropy-driven effective growth forces, to be determined either theoretically or phenomenologically according to the system under study. Then, from equation (2), the newly proposed form for the effective parameter ${\rm{\Phi }}({D}_{0},\zeta )$ allows us to define a general dimensionality function $D({D}_{0},\zeta )$, characterized by an inflection point ${\zeta }_{{\rm{i}}}$, associated with a regime change in growth dynamics that satisfies ${\rm{\Lambda }}{\zeta }_{{\rm{i}}}^{\chi }/{D}_{0}=(\chi -1)/\chi$. Additionally, from equation (3), the inflection point for the first-order approximation ${D}^{(1)}({D}_{0},\zeta )$ is characterized by ${\rm{\Lambda }}{\zeta }_{{\rm{i}}}^{\chi }/{D}_{0}=(\chi -1)/(\chi +1)$. In this way, the expressions for the generalized dimensionality function $D({D}_{0},\zeta )$ describe the scaling of the clusters along a continuous morphological transition from *D* = *D*_0_ for ${\rm{\Phi }}(\zeta )=0$ towards *D* = 1 as ${\rm{\Phi }}(\zeta )\to \infty$, with a well-defined regime change in growth dynamics at ${\zeta }_{{\rm{i}}}$. In the following, in order to test our model, we will apply it to two morphological transitions, namely DLA-MF and BA-MF, newly developed for this work. Then, we will address the DBM once more, aiming to develop a possible solution to its fractality. Finally, we will discuss the universal scaling presented by these systems.

### DLA/BA-MF morphological transitions

**Figure 2:**
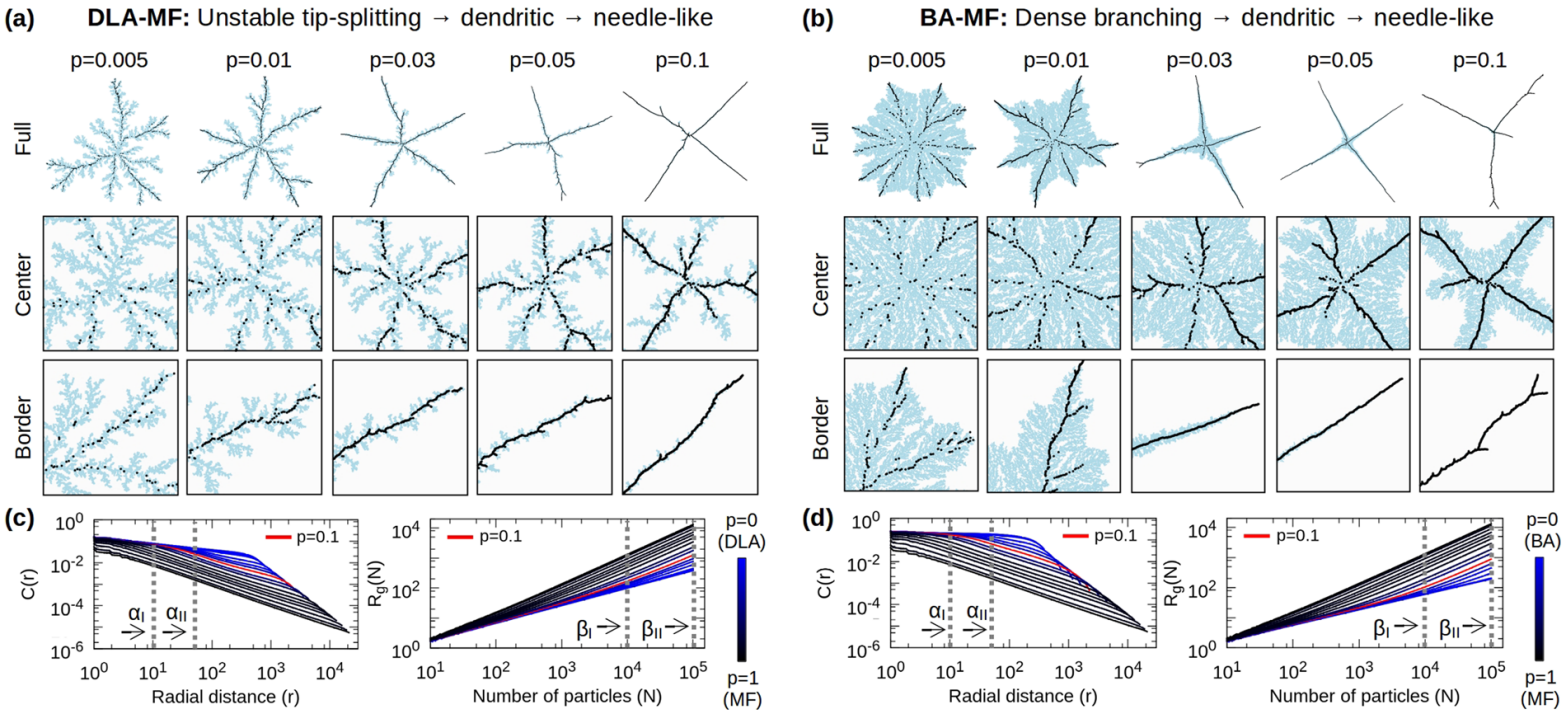
DLA/BA-MF Transitions. Clusters of 1.5 × 10^5^ particles grown with the indicated values of *p* are shown at different magnifications for the (**a**) DLA-MF and (**b**) BA-MF transitions. Particles aggregated under DLA/BA are coloured in light-blue while those through MF in black. These transitions exhibit fast morphological transformations as *p* increases, from unstable tip-splitting (DLA) or dense branching (BA), through (inhomogeneous) dendritic, to needle-like growth (MF). (**c**,**d**) *C*(*r*) and *R*_*g*_(*N*) display deviations from a well-defined linear behaviour for different values of *p*, revealing the inhomogeneity or crossover effects in these clusters. This is better appreciated at low scales, where the stochasticity of DLA or BA dominates the local growth, whereas MF tends to dominate the global morphology as *p* → 1. In both cases, the dynamical growth-regime changes at $p\approx 0.1$. Labels *α*_*I*_, *α*_*II*_, *β*_*I*_ and *β*_*II*_ indicate the regions used for the scaling analysis.

In *d* = 2, a general stochastic aggregation process can be modeled under a Monte Carlo scheme, involving three fundamental and simple *off*-*lattice* models of particle-cluster aggregation. On the one hand, the well-known BA and DLA models will provide disordered/fractal structures by means of their pure stochastic dynamics (Fig. 1a,b). On the other, a mean-field (MF) model of long-range interactive particle-cluster aggregation^27, 30^, will provide the energetic dynamical element (see Fig. 1c). The statistical combination of these models results in off-lattice DLA-MF and BA-MF dynamics, whose morphological transitions can be controlled by the mixing parameter $p\in [\mathrm{0,}\,1]$, associated with the fraction of particles aggregated under MF dynamics, $p={N}_{{\rm{MF}}}/N$, where *N* is total number of particles in the cluster. In this way, as *p* varies from *p* = 0 to *p* = 1, it generates two non-trivial transitions from fractals (DLA) or fat-fractals (BA) with respective *D*_0_, to non-fractal clusters with *D* = 1 (MF), that capture all the main morphologies of fractal growth^6^ (see Fig. 2). In the following, the data for *D* was measured and averaged over an ensemble of 128 clusters (with *N* = 1.5 × 10^5^ particles), for each value of *p*, by means of two standard methods: the two-point density correlation function, $C(r)\propto {r}^{-\alpha }$, and the radius of gyration, ${R}_{g}(N)\propto {N}^{\beta }$, where the scaling exponents are related to *D* as *D*_*α*_ = *d* − *α* and *D*_*β*_ = 1/*β*, respectively (see Methods).

**Figure 3:**
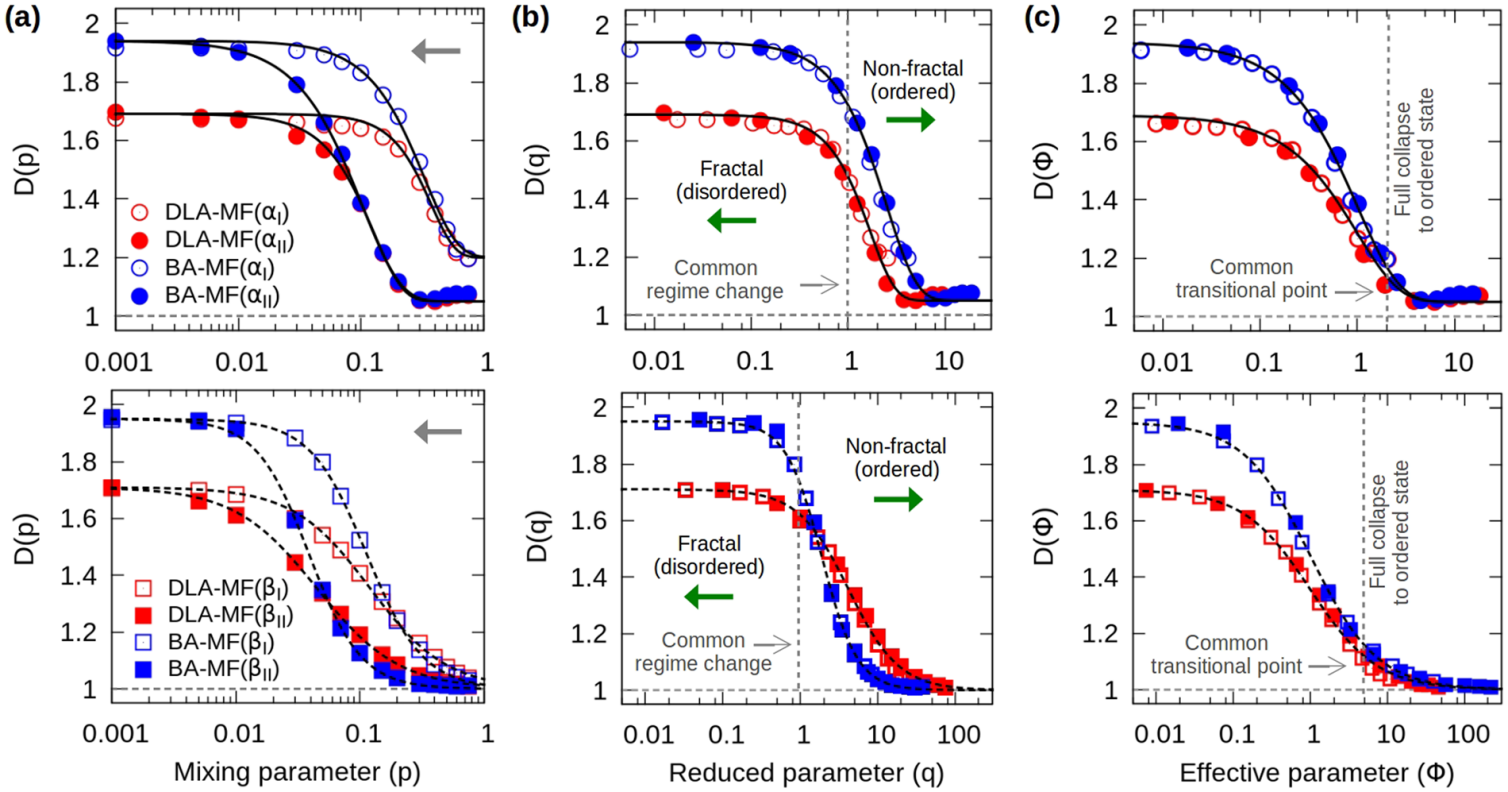
Scaling analysis of the BA/DLA-MF transitions. (**a**) Local scaling, *D*(*p*), of the DLA-MF and BA-MF transitions obtained from *C*(*r*) [top], at small (*α*_*I*_) and large (*α*_*II*_) scales, and *R*_*g*_(*N*) [bottom], at medium (*β*_*I*_) and large (*β*_*II*_) scales, according to Fig. 2c,d (see Methods). These results are described by the solid and dotted curves given by equation (2) [top] and (3) [bottom], respectively, for different values of the parameters ${\rm{\Lambda }}$ and *χ*. The arrows indicate the direction of increasing scale. (**b**) By plotting *D* as a function of *q* = *p*/*p*_i_ (where *p*_i_ is calculated for each curve), all the data collapse into single master curves, *D*(*q*), according to equations (2) [top] and (3) [bottom], with a common point of regime change at *q*_i_ = 1. The arrows indicate the direction in which the fractal or non-fractal features of the clusters are enhanced. (**c**) Plots of *D* as a function of the effective parameter ${\rm{\Phi }}$. Notice how all of the transitions approach common transitional points where clusters have already fully collapsed to linear $(D\approx 1)$ structures, independently of the stochastic model used. Details of the values for the parameters used can be found in Table 2.

In contrast to the BA-DLA or DBM transitions, the DLA-MF and BA-MF transitions are characterized by inhomogeneous clusters, i.e., structures with non-constant scaling (see Fig. 2c,d). Given this *multiscaling* or crossover behaviour, it is not possible to strictly define their fractal dimensions for intermediate values of *p*, because the measurements are scale dependent. Nonetheless, these multiscaling features can be properly quantified by measuring a local scaling or “local fractal dimension”, *D*(*p*), at different scales^7^ (see Fig. 3a). In this particular case, the selection of the scales for the analysis was made with the purpose of making evident the change of slope of the *C*(*r*) and *R*_*g*_(*N*) functions at short and long scales (see Methods).

In particular, it should be noted that all of the measurements for *D*(*p*) display a clear behaviour from *D*_0_ for *p* = 0 to *D* = 1 for *p* = 1, with the exception of the data for *D*(*p*) obtained from *C*(*r*) at small scales, that does not fully collapse to one as *p* → 1 (see data labelled as *α*_*I*_ in the upper panel of Fig. 3a). This feature can be associated with the locality of the measurements with *C*(*r*), that samples over many different origins and, therefore, captures more details of the fine structure of the cluster. In contrast, measurements performed with *R*_*g*_(*N*) have a common fixed origin (the seed particle), thus providing a coarser description (see Methods). Then, at small scales, when clusters seem to be more compact (“less” fractal), i.e., when finite-size effects are inevitable, the limitations of the respective quantities are exhibited in the computation of *D*. Nonetheless, this feature does not compromise the reliability of the simulations results nor the analytical analysis performed over the data as we show below.

## Discussion

Analytically, all the measurements of *D*(*p*) for the DLA-MF and BA-MF transitions can be described by equations (2) and (3), by identifying $\zeta =p$ in the expression for the general *ansatz* ${\rm{\Phi }}({D}_{0},\zeta )={\rm{\Lambda }}{\zeta }^{\chi }/{D}_{0}$, with *D*_0_ being the fractal dimension of the BA or the DLA cluster obtained when *p* = 0, and by using ${\rm{\Lambda }}$ and *χ* as fitting parameters. Remarkably, we found that the data for *D*(*p*), as obtained through *C*(*r*), is very well described by equation (2), whereas equation (3) describes best the results obtained through *R*_*g*_(*N*) (see Fig. 3a). As previously discussed, even though the data for *D*(*p*) obtained through *C*(*r*) at small scales does not fully collapse to one $(D\approx 1.2)$ as *p* → 1 (see the curves with subscript *α*_*I*_ in the upper panel of Fig. 3a), the functional form of ${\rm{\Phi }}({D}_{0},p)$ allows for these cases to be considered as, for *p* = 1, we have ${\rm{\Phi }}({D}_{0}\mathrm{,1})={\rm{\Lambda }}/{D}_{0}$. Indeed, a condition for the full collapse to *D* = 1 for *p* = 1 would require ${\rm{\Lambda }}/{D}_{0}\gg 1$, condition that is currently satisfied by the BA-MF and DLA-MF transitions (see Table 2).

**Table 2:** Descriptions based on *D*(*p*) and *D*(*q*).

Transition	Method	Scale	Λ	*χ*	*D*_0_	*p*_i_	*q*_i_
DLA-MF	*C*(*r*)	*α*_*I*_	15.4	2.24	1.6749 ± 0.0024	0.29	
		*α*_*II*_	71.5	1.82		0.08	
	*R*_*g*_(*N*)	*β*_*I*_	33.8	1.41	1.7100 ± 0.0007	0.03	
		*β*_*II*_	101.6	1.32		0.01	
BA-MF	*C*(*r*)	*α*_*I*_	11.6	1.61	1.9384 ± 0.0001	0.18	
		*α*_*II*_	45.4	1.38		0.04	
	*R*_*g*_(*N*)	*β*_*I*_	124.8	1.95	1.9485 ± 0.0001	0.06	
		*β*_*II*_	1547.7	2.05		0.02	
DLA-MF	*C*(*r*)			1.69	1.6749 ± 0.0024		1.0
	*R*_*g*_(*N*)			1.34	1.7100 ± 0.0007		1.0
BA-MF	*C*(*r*)			1.39	1.9384 ± 0.0001		1.0
	*R*_*g*_(*N*)			1.88	1.9485 ± 0.0001		1.0
DBM (numerical)			0.70	1.26	2.0	0.66	
DBM (theoretical)			0.685	1.52	2.0	1.0	1.0

First section: parameter values used to describe *D*(*p*, Λ, *χ*), using equations (2) and (3). In order to determine the inflection points, $({\rm{\Lambda }}/{D}_{0}){p}_{{\rm{i}}}^{\chi }=(\chi -1)/\chi$ for the measurements obtained through *C*(*r*), while $({\rm{\Lambda }}/{D}_{0}){p}_{{\rm{i}}}^{\chi }=(\chi -1)/(\chi +1)$ for those obtained through *R*_*g*_(*N*). Second section: equations (2) and (3), as function of *q* = *p*/*p*_i_, are used to fit the data for *D*(*q*, *χ*) obtained through *C*(*r*) and *R*_*g*_(*N*), respectively. In this description, only *χ* is remains as a fitting parameter and, by construction, all the inflection points are located at *q*_i_ = 1. Third section: results for the DBM obtained through equation (2).

Furthermore, our model allows us to determine the corresponding inflection point *p*_i_ for each curve *D*(*p*), however, due to the inhomogeneity of the clusters and the measurements’ scale dependence, it is impossible to establish a unique regime-change point for each curve in terms of *p*. Nonetheless, such description can still be achieved by introducing the *reduced* parameter $q=p/{p}_{{\rm{i}}}$, under which, all the data for *D*(*p*) collapse into single curves *D*(*q*), according to their respective transition (DLA-MF or BA-MF). Analytically, substituting $q\in \mathrm{[0},\infty )$, back into equation (2), we have that ${\rm{\Phi }}(q)={q}^{\chi }(\chi -1)/\chi$ and, for equation (3), the effective parameter is now given by ${\rm{\Phi }}(q)={q}^{\chi }(\chi -\mathrm{1)/(}\chi +\mathrm{1)}$. In this framework, the variations in $D(p,{\rm{\Lambda }},\chi )$ due to crossover effects disappear and each transitions is now described by a single master curve *D*(*q*, *χ*). Also, by construction, the dynamical growth-regime change is now located at *q*_i_ = 1 for all transitions. By fitting *D*(*q*, *χ*) given by equations (2) and (3) to the respective data, we obtain again an excellent agreement with the numerical results (see Fig. 3b).

**Figure 4:**
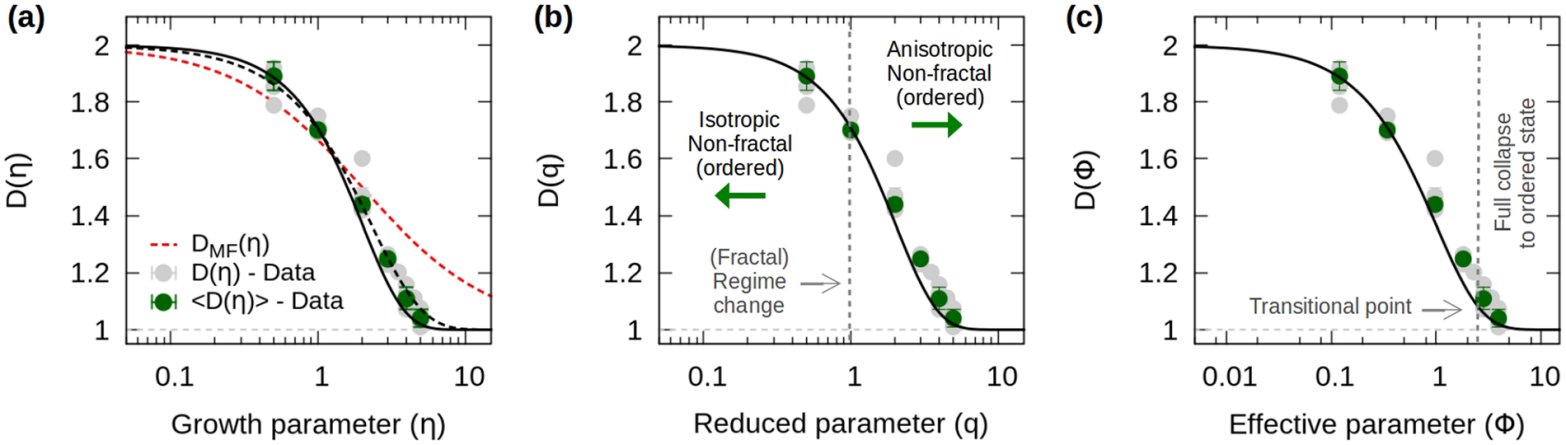
Scaling analysis of the DBM transition. (**a**) Plot of the fractal dimensions *D* as a function of *η* for the two-dimensional DBM transition (see Table 1). Here, the failure of the mean-field description is evident (dashed red curve). A better agreement is obtained using equation (2), with ${\rm{\Phi }}({D}_{0},\eta )={\rm{\Lambda }}{\eta }^{\chi }/{D}_{0}$, where *D*_0_ = 2. By fitting the data with this equation, one obtains ${\rm{\Lambda }}\approx 0.70$ and $\chi \approx 1.26$ with ${\eta }_{i}\approx 0.66$ (dashed black curve), while from the analytical analysis and by considering *η*_*i*_ = 1, ${\rm{\Lambda }}=-2\,\mathrm{log}\,(0.71)\approx 0.685$ and $\chi =1/(1-{\rm{\Lambda }}/{D}_{0})\approx 1.52$ (solid black curve). (**b**) Maintaining *η*_i_ = 1 as the transitional point, the analytical solution for *D*(*q*) is equivalent to that for *D*(*η*). (**c**) As well, from the analytical expressions, one can obtain the curve $D({\rm{\Phi }})$ (see Table 3).

At this point in the analysis, it is important to consider the two-dimensional DBM transition as well. As previously discussed, within the mean-field approximation, we have that equation (3), with ${{\rm{\Phi }}}_{MF}=\eta /d$ (*d*_*w*_ = 2), fails to precisely describe the fractality of the transition (see dashed red curve in Fig. 4a). However, by means of equation (2) and the general *ansatz*, ${\rm{\Phi }}({D}_{0},\eta )={\rm{\Lambda }}{\eta }^{\chi }/{D}_{0}$, with *D*_0_ = *d*, a better agreement with the data is achieved. The parameters ${\rm{\Lambda }}$ and *χ* can be obtained by fitting our model to the data as before (dashed black curve in Fig. 4a), nonetheless, here we also show how they can be analytically calculated. Setting *d* = 2, the first parameter ${\rm{\Lambda }}$ can be obtained by using the well known result for the two-dimensional scaling of DLA, *D* = 1.71, that is associated with *η* = 1 for the DBM. From equation (2), this leads to ${\rm{\Lambda }}=-d\,\mathrm{log}\,(({D}_{\eta =1}-1)/(d-1))=-2\,\mathrm{log}\,(0.71)\approx 0.685$. Then, the parameter *χ* is obtained from the dynamical condition imposed over *D*, given by ${\rm{\Lambda }}{\eta }_{i}^{\chi }/d=(\chi -1)/\chi$. Considering that the DLA fractal (*η* = 1) can be associated to a particular (possibly critical^28, 29^) dynamical state, that defines the regime change in the DBM transition, from non-fractal (*D*_0_ = *d*, *η* = 0), through fractal (DLA, *η* = 1), to non-fractal (*D* = 1, $\eta \gg 1$), we can set *η*_*i*_ = 1, leading to $\chi =1/(1-{\rm{\Lambda }}/d)\approx 1.52$. As it can be appreciated in Fig. 4 (solid black curves), this analytical result agrees very well with the data for *D*(*η*) within a self-contained framework, provided that the DLA state marks the point of change in regime. For the rest of the article we will consider *η*_*i*_ = 1 as the transitional point for the DBM.

An important issue to consider here is that of the criticality of these morphological transitions, as well as its characterization using the fractal dimension as an order parameter, as previously suggested for the DBM^21^. In order to address this point in a comprehensive approach, let us first define a possible and suitable order parameter for these systems. This is done by plotting all of the data for *D*(*q*) now as function of ${\rm{\Phi }}$ itself, i.e., $D({\rm{\Phi }})$, depicted in Figs 3c and 4c. Notice that, in this description, the DLA/BA-MF (Fig. 3c) and DBM (Fig. 4c) transitions, starting from *D*_0_, approach the highly anisotropic regime $(D\approx 1)$ in an almost identical manner, in excellent agreement with equations (2) and (3). Further on, in order to remove the dependence on *D*_0_, we introduce the reduced co-dimension, ${D}^{\ast }\in [\mathrm{0,\; 1}]$, defined by ${D}^{\ast }=(D-1)/({D}_{0}-1)$, as the new “order parameter” of the system. From equations (2) and (3), we respectively have,

4 $${D}^{\ast }({\rm{\Phi }})={e}^{-{\rm{\Phi }}},\,{D}^{\ast (1)}({\rm{\Phi }})=\frac{1}{1+{\rm{\Phi }}}\mathrm{.}$$

**Figure 5:**
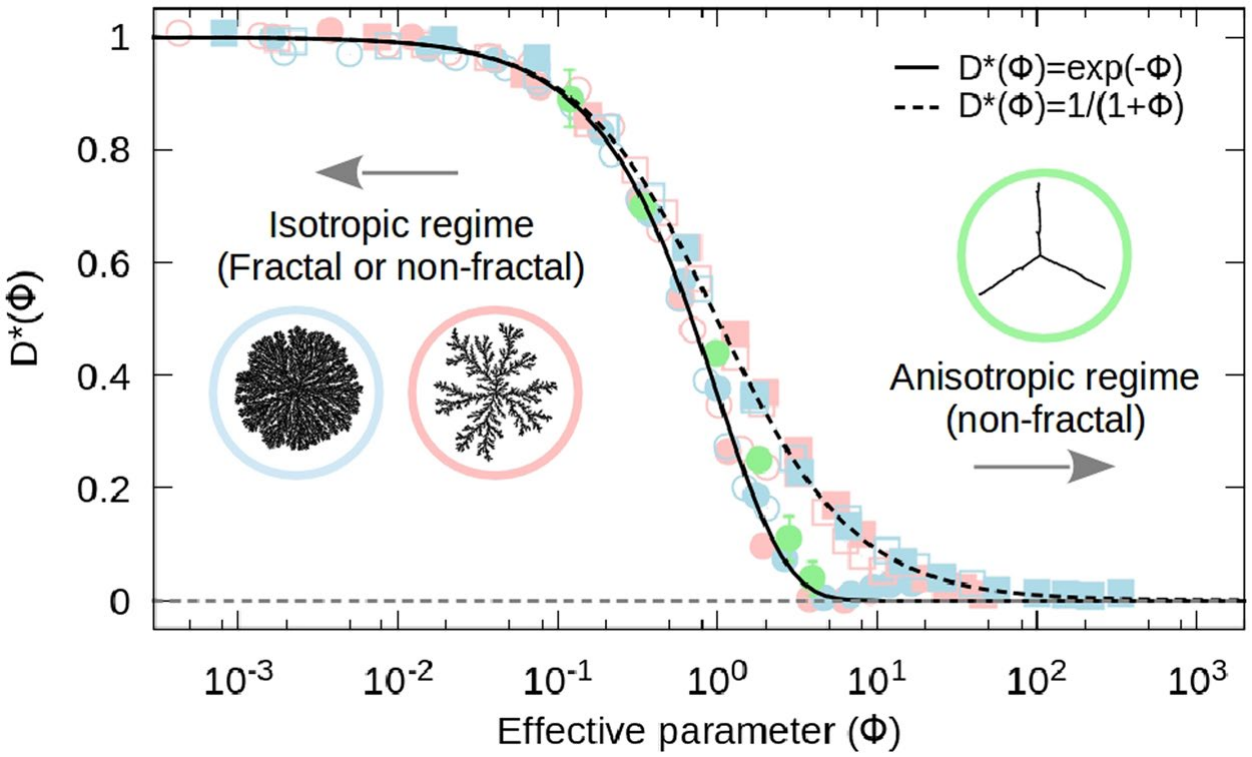
Universality. By plotting the reduced co-dimension D* as function of the effective parameter ${\rm{\Phi }}$, data for the scaling of the DLA-MF, BA-MF and DBM transitions collapse to universal curves given directly by equations (2) and (3). Under this description, these universal morphological transitions are independent of the fractal dimension of the initial configuration, *D*_0_, the symmetry-breaking process that drives the transition (including crossover effects), and, even, independent of the Euclidean dimension *d* of their embedding space. The scattered points were obtained by processing the data of Figs 3 and 4.

In this manner, under the new framework based on the co-dimension D*, all the numerical results collapse into the universal curves given by equations (4) as can be appreciated in Fig. 5. These curves go from D* = 1 for *D* = *D*_0_, to D* → 0 as *D* → 1. Moreover, the co-dimension D* is not necessarily describing a real “order-disorder” transition but, rather, an isotropic-anisotropic one. The subtlety lies at the initial cluster configuration. This is, even though all transitions collapse to a linear “ordered” structure, the initial cluster configuration can also be considered as ordered, such as in the case of the DBM (associated to compact Eden clusters), or disordered, as in the case of the BA/DLA-MF transitions (a fractal for DLA and a fat-fractal for BA). Nonetheless, in terms of their isotropy, or preferential growth features, all transitions start from an isotropic (such as Eden or BA) or isotropic on-average (such as DLA) clusters, to a highly anisotropic structure as the rotational-symmetry brakes down.

Even more, given that in any case the solutions for D* are smooth functions that tend to zero in a continuous manner, defining an specific point where D* becomes exactly zero is not possible. This implies that the previously suggested critical point for the DBM, i.e., the value for *η* where $D\approx 1$ ^21^, cannot be treated as “critical” from the point of view of a formal critical phase-transition theory^28, 29^. In fact, this will not be possible for any of the transitions analyzed in this work. Nevertheless, what it is still possible is to define *transitional* points, ${{\rm{\Phi }}}_{t}$, that are different from the points where the growth-regime changes. For the transitional points, the screening/anisotropy effects strongly dominate the morphology of the cluster, thus, they correspond to points at which $D=1+\varepsilon$, with $\varepsilon \ll 1$ is the tolerance or deviation from *D* = 1 (for technical details see Table 3).

**Table 3:** Transitional points.

Transition	Method	Parameter	*D*_0_	*χ*	Φ_*t*_(*ε* = 0.1)	*q*_*t*_	Φ_*t*_(*ε* = 0.05)	*q*_*t*_	*D*(*q* = 1)
DLA-MF	*C*(*r*)	*p*	1.6749 ± 0.0024	1.69	2.3	2.8	3.0	3.2	1.46 ± 0.02
DLA-MF	*R*_*g*_(*N*)	*p*	1.7100 ± 0.0007	1.34	9.0	21.7	19.0	37.8	1.62 ± 0.01
BA-MF	*C*(*r*)	*p*	1.9384 ± 0.0001	1.39	2.3	4.5	3.0	5.4	1.72 ± 0.02
BA-MF	*R*_*g*_(*N*)	*p*	1.9485 ± 0.0001	1.88	9.0	6.0	19.0	9.0	1.73 ± 0.01
DBM (theoretical)		*η*	2.0	1.52	2.3	3.5	3.0	4.2	1.71

By considering $(D({{\rm{\Phi }}}_{t})-1)/({D}_{0}-1)=\varepsilon$, where $\varepsilon \ll 1$ is the tolerance or deviation from *D* = 1 (see Fig. 3c), the *universal* transitional points, ${{\rm{\Phi }}}_{t}$, must satisfy, $\exp \,(-{{\rm{\Phi }}}_{t})=\varepsilon$ and ${{\rm{\Phi }}}_{t}=(1-\varepsilon )/\varepsilon$, from equations (2) and (3), respectively. In order to recover the particular transitional points, we must recall that ${{\rm{\Phi }}}_{t}={\rm{\Phi }}({q}_{t})$ and solve for *q*_*t*_. Notice also that ${q}_{t}={q}_{t}(\varepsilon ,\chi ,{D}_{0})$, therefore, it yields different values for each transition. Additionally, we must point out that the strong similarities exhibited by the BA-MF and DBM transitions, such as ${q}_{{\rm{t}}}\approx 4$ and $D\approx 1.71$ for *q* = 1 (similar to *η* = 1), even though we are dealing with completely different processes.

The final and most important implication of the previous findings is that the DBM and BA/DLA-MF transitions, although completely different, can be treated as belonging to the same universality class. In order to make sense of this, we must recall that, in two-dimensions, the DBM (for *η* = 1) and viscous fingering phenomena are said to belong to the same universality class as that of DLA, based on the fact that they are all characterized by the same fractal dimension, *D* = 1.71^12, 31^. Therefore, by extending this idea to a whole set of dimensions, the *universality* of these morphological transitions must be understood in the sense that they are all described by the same set of fractal dimensions. Quite remarkably, under the description provided by the co-dimension ${D}^{\ast }({\rm{\Phi }})$, the DBM and BA/DLA-MF morphological transitions belong to the same universality class which, in turn, implies that their mathematical description is independent of their spatial symmetry-breaking dynamics and initial configuration, therefore, these transitions will be described by the same curves in any embedding Euclidean dimension (see Fig. 5).

In summary, we present a novel framework for the scaling of morphological transitions in stochastic growth processes. By means of a general *ansatz* for an effective control parameter, ${\rm{\Phi }}$, we were able to construct a model for the fractal dimension *D* that is able to describe the fractality of very different systems. In particular, this model is able to describe the scaling of the newly introduced BA/DLA-MF transitions, as well as it provides an excelent description for the fractal dimensions of the well-known DBM. In addition, it was strictly shown that *D* can be used as a rotational-symmetry “order” parameter under the reduced co-dimension transformation D*. On the other hand, we have shown that the previously suggested “critical” point for the DBM cannot be properly defined as such, but instead, as a transitional point in the fractality of a continuous morphological transition. Finally, we have shown that under the reduced co-dimension, the DLA-MF, BA-MF and DBM transitions exhibit a well-defined universal scaling, ${D}^{\ast }({\rm{\Phi }})$, that is remarkably independent of their initial configuration, the specific spatial symmetry-breaking mechanism that drives the transition, and the dimensionality of their embedding Euclidean space. In general, we consider that the results and models presented in this work represent a significative unifying step towards a complete scaling theory of fractal growth and far-from-equilibrium pattern formation. Additionally, the possibility of applying this model to discuss current issues in fractal growth-phenomena and other related research areas, ranging from biology^1, 3^, intelligent materials engineering^32, 33^ to medicine^34–36^, seems to be more feasible and direct.

## Methods

### Aggregation dynamics

In all simulations, each particle has a diameter equal to one. This is the basic unit of distance of the system. For aggregates based on BA or MF (Fig. 1a,c), we follow a standard procedure in which particles are launched at random from a circumference of radius *r*_*L*_ = 2*r*_*max*_ + *δ*, with equal probability in position and direction of motion. Here, *r*_*max*_ is the distance of the farthest particle in the cluster with respect to the seed particle placed at the origin. In our simulations we used *δ* = 1000 particle diameters to avoid undesired screening effects. For the MF model, particles always aggregate to the closest particle in the cluster with respect to their incoming path. This is determined by the projected position of the aggregated particles along the direction of motion of the incoming particle (see Fig. 1c). In the case of aggregates based on DLA (Fig. 1b), particles were launched from a circumference of radius *r*_*L*_ = *r*_*max*_ + *δ*, with *δ* = 100. The mean free path of the particles is set to one particle diameter. We also used a standard scheme that modifies the mean free path of the particles as they wander at a distance larger than *r*_*L*_ or in-between branches, as well as the common practice of setting a killing radius at *r*_*K*_ = 2*r*_*L*_ in order to speed up the aggregation process.

In order to mix different aggregation dynamics, a Monte Carlo scheme of aggregation is implemented using the BA, DLA and MF models. The combination between pairs of models results in the DLA-MF and BA-MF transitions, controlled by the mixing parameter $p\in [0,1]$, associated with the probability or fraction of particles aggregated under MF dynamics, $p={N}_{{\rm{MF}}}/N$, where *N* is total number of particles in the cluster. Therefore, as *p* varies from *p* = 0 (pure stochastic dynamics given by the BA or DLA models) to *p* = 1 (purely energetic dynamics given by the MF model), it generates the two transitions introduced in the work. The evaluation of the aggregation scheme to be used is only updated once a particle has been successfully aggregated to the cluster under such dynamics.

### Fractal and scaling analysis

In all of the measurements, we used 128 clusters containing 1.5 × 10^5^ particles. Formally, the fractal dimension is measured from the two-point density correlation function, $C(r)={\langle \langle \rho ({{\bf{r}}}_{0})\rho ({{\bf{r}}}_{0}+{\bf{r}})\rangle \rangle }_{|{\bf{r}}|=r}$, where the double bracket indicates an average over all possible origins **r**_0_ and all possible orientations. For this work, we made use of 1000 possible origins. Here, it is assumed that $C(r)\approx {r}^{-\alpha }$, where the fractal dimension is given by *D*_*α*_ = *d* − *α*, where *d* is the dimension of the embedding space. We also used the radius of gyration given by ${R}_{g}^{2}={\sum }_{i=1}^{N}\,{({{\bf{r}}}_{i}-{{\bf{r}}}_{CM})}^{2}$, where *N* is the number of particles, **r**_*i*_ is the position of the *ith*-particle in the cluster, and **r**_*CM*_ is the position of the center of mass. In this scheme, it is assumed that ${R}_{g}(N)\approx {N}^{\beta }$, where the fractal dimension is given by *D*_*β*_ = 1/*β*. Therefore, the fractal dimensions, *D*_*α*_ and *D*_*β*_, are respectively obtained from linear-fits to the corresponding functions, *C*(*r*) and *R*_*g*_(*N*), in log-log plots for different scales.

In practice, it is assumed that *α* and *β* are constant as long as the size or number of particles in the cluster is large enough. However, because the clusters do not develop a constant scaling, linear-fits at different scales were performed in order to capture their main local fractal features. Also, we averaged the outcome of 10 linear fits, distributed over a given interval in order to improve the precision of the measurements. For both transitions, DLA-MF and BA-MF, *D*_*α*_(*p*) is measured at short length-scales (this is *α*_*I*_) over the interval ${r}_{i}\in [1,2]$ with fitting-length equal to 10, and ${r}_{f}\in [11,12]$ (in particle diameters units). At long length-scales (*α*_*II*_), over ${r}_{i}\in \mathrm{[10},\mathrm{11]}$ with fitting-length equal to 40, and ${r}_{f}\in [50,51]$. On the other hand, for *D*_*β*_(*p*), the measurements at medium scales (*β*_*I*_) where performed over the interval ${r}_{i}\in [{10}^{2},{10}^{3}]$ with fitting-length equal to 10^4^ and ${r}_{f}\in [1.01\times {10}^{4},1.1\times {10}^{4}]$ (in particle number), while, at large scales (*β*_*II*_), over the interval ${r}_{i}\in [{10}^{3},{10}^{4}]$ with fitting-length equal to 0.9 × 10^5^ and ${r}_{f}\in [9.1\times {10}^{4},{10}^{5}]$.
